# Quantification of joint blood flow by dynamic contrast-enhanced near-infrared spectroscopy: application to monitoring disease activity in a rat model of rheumatoid arthritis

**DOI:** 10.1117/1.JBO.25.1.015003

**Published:** 2020-01-14

**Authors:** Seva Ioussoufovitch, Laura B. Morrison, Lise Desjardins, Jennifer A. Hadway, Keith St. Lawrence, Ting-Yim Lee, Frank Beier, Mamadou Diop

**Affiliations:** a Western University, Bone and Joint Institute, School of Biomedical Engineering, Faculty of Engineering, London, Ontario, Canada; b Lawson Health Research Institute, Imaging Program, London, Ontario, Canada; c Western University, Schulich School of Medicine and Dentistry, Department of Medical Biophysics, London, Ontario, Canada; d Robarts Research Institute, Imaging Program, London, Ontario, Canada; e Western University, Schulich School of Medicine and Dentistry, Department of Physiology and Pharmacology, London, Ontario, Canada

**Keywords:** time-resolved near-infrared spectroscopy, dynamic contrast-enhanced, blood flow, rheumatoid arthritis, treatment monitoring, disease-modifying antirheumatic drugs

## Abstract

**Significance**: Current guidelines for rheumatoid arthritis (RA) management recommend early treatment with disease modifying antirheumatic drugs (DMARDs). However, DMARD treatment fails in 30% of patients and current monitoring methods can only detect failure after 3 to 6 months of therapy.

**Aim**: We investigated whether joint blood flow (BF), quantified using dynamic contrast-enhanced time-resolved near-infrared spectroscopy, can monitor disease activity and treatment response in a rat model of RA.

**Approach**: Ankle joint BF was measured every 5 days in eight rats with adjuvant-induced arthritis (AIA) and four healthy controls. Arthritis was allowed to progress for 20 days before rats with AIA were treated with a DMARD once every 5 days until day 40.

**Results**: Time and group had separate significant main effects on joint BF; however, there was no significant interaction between time and group despite a notable difference in average joint BF on day 5. Comparison of individual blood flow measures between rats with AIA and control group animals did not reveal a clear response to treatment.

**Conclusions**: Joint BF time courses could not distinguish between rats with AIA and study controls. Heterogeneous disease response and low temporal frequency of BF measurements may have been important study limitations.

## 1 Introduction

Rheumatoid arthritis (RA) is a chronic and progressive autoimmune disease that afflicts about 1% of the population and is associated with pain,^1^ reduced quality of life,^2^ and disability.^3^ Fortunately, the introduction of a treatment paradigm that combines treat-to-target strategies^4^ with early use of disease-modifying antirheumatic drugs (DMARDs)^5^ has significantly improved mid- and long-term patient outcomes over the past two decades. However, DMARD treatment failure, which is currently identified only after 3 to 6 months of therapy, still occurs in 30% of RA patients.^6^^,^^7^ After failure, these patients must undergo an iterative process where they are assigned to new therapies and wait again for 3 to 6 months before treatment efficacy can be reliably assessed. This process is drawn-out, costly, and results in patients losing the benefits of effective early treatment while having an increased risk of developing irreversible joint damage.^8^

Treatment response is currently assessed using a combination of clinical examination and patient self-assessment. Though this approach is the current standard of care, it can be ineffective for repetitive, longitudinal assessments because of its subjectivity and variability.^9^^,^^10^ Furthermore, clinical examination and patient self-assessment are unlikely to detect subclinical changes in inflammation, which could be early indications of treatment response. As such, clinical examination is often supplemented with laboratory tests, radiography, or ultrasonography. However, though these additional tools can be effective at aiding diagnosis, their usefulness for long-term monitoring is limited by low sensitivity (radiography and laboratory tests)^11^ and suboptimal reproducibility (ultrasonography).^12^^–^^14^ Given the above limitations, there is currently a need to find more sensitive and objective techniques that can detect RA treatment failure within the first 3 months of therapy.

In recent years, there has been a growing interest in investigating the ability of near-infrared (NIR) optical methods to assess RA disease progression. This is in part due to the ability of NIR techniques to provide quick and objective measurements at a relatively low cost. In the case of RA, NIR methods generally attempt to monitor disease progression by measuring downstream effects of joint hypoxia, which is well known to play a central role in the maintenance and progression of the disease.^15^^,^^16^ It is well established that chronic inflammation, which is a key feature of RA, causes hypertrophy of the synovial lining; the thickness of the synovial membrane increases from 1 to 3 to over a dozen cell layers in RA.^16^ This hypertrophic cell mass induces an increased metabolic demand that exceeds supply, leading to the development of hypoxic regions within the synovium.^15^^,^^17^ In fact, it has been shown (using invasive probes) that the partial pressure of oxygen is lower in inflamed joints than in healthy joints.^18^ The presence of chronic hypoxia then acts as a signal both for the formation of new blood vessels (i.e., angiogenesis)^17^ and increased tissue blood flow (BF).^19^^,^^20^

Previous studies using NIR techniques have largely focused on assessing changes in oxygen saturation and blood content within the joint since these characterize hypoxia and angiogenesis, respectively, and are the downstream effects of chronic inflammation. Some examples of NIR techniques that have been applied to RA disease monitoring include photoacoustic tomography,^21^^–^^24^ diffuse optical spectroscopy,^25^^–^^27^ contrast-based fluorescence imaging,^28^^–^^30^ and diffuse optical tomography.^31^^–^^34^ Using these approaches, RA disease progression can be assessed by measuring increases in blood volume, variations in blood oxygen saturation, intensity of contrast agent fluorescence, and total hemoglobin concentration in the joint. As mentioned, increased tissue BF—defined as the volume of blood flowing through a mass of tissue per unit time (mL/min/100 g)—is another physiological response to chronic hypoxia; in fact, joint BF has yet to be thoroughly investigated as a potentially highly sensitive marker of RA disease activity. Thus, we hypothesized that BF can be a surrogate marker of changes in joint hypoxia and RA disease progression. We previously developed a dynamic contrast-enhanced time-resolved near-infrared spectroscopy (DCE TR-NIRS) technique for measuring joint BF and showed—in a rabbit model of RA—that joint BF is more sensitive to inflammatory arthritis than changes in hemoglobin concentration and oxygen saturation.^35^ These findings suggest that joint BF may be a more sensitive biomarker of inflammatory arthropathies than those previously investigated with other NIR techniques. Thus, the objective of this work was to investigate whether joint BF, as measured with DCE TR-NIRS, can track longitudinal changes in joint inflammation during disease induction and DMARD treatment in a rat model of RA.

## 2 Methods

### 2.1 Instrumentation

The TR-NIRS system was built in-house using a pulsed diode laser (LDH-P-C-810; PicoQuant, Germany) connected to a PDL 828 laser driver (PicoQuant). The laser emission was centered at 805 nm and the pulse repetition rate was set to 80 MHz. The laser output was coupled into an emission fiber (ϕ=400  μm, NA=0.22; Fiberoptics Technology, Pomfret, Connecticut) that guided light to the ankle joint. Light transmitted through the ankle joint was collected with a fiber optic bundle (ϕ=3  mm, NA=0.55; Fiberoptics Technology) that was coupled to a hybrid photomultiplier detector (PMA Hybrid; PicoQuant) and a time-correlated single photon-counting module (HydraHarp 400; PicoQuant). A three-dimensional (3-D)-printed probe-holder was used to ensure good contact between the ankle joint and the emission and detection probes. Instrument response functions (IRFs) were acquired by placing the emission and detection probes into a light-tight box containing a piece of paper, positioned 2 mm away from the emission fiber, which acted as a light diffuser to fill the numerical aperture of the detection probe.

### 2.2 Phantom Experiments

Since the DCE TR-NIRS technique relies on accurate estimation of changes in tissue absorption, we assessed the system’s ability to measure absorption changes in a tissue-mimicking phantom made with Intralipid and India Ink. A cuvette (10  mm×10  mm×40  mm) was filled with 3.5 mL of 0.8% Intralipid created by diluting a 20% stock solution of Intralipid with water. We then placed the emission and detection probes transversely across the cuvette, secured the entire setup using a 3-D-printed holder, and acquired a baseline measurement with the TR-NIRS system. Next, we began adding 0.02-mL increments of an India Ink solution with a known absorption coefficient ($\mu_{a,\mathrm{ink}}$). Note that $\mu_{a,\mathrm{ink}}$ was determined by measuring light transmittance through an India ink dilution to determine the ink’s molar absorption coefficient (see Sec. 2.4 for details). The volume of increments was chosen so that the investigated changes in absorption would cover a range similar to what had been measured during preliminary animal experiments (0.005 to $0.040\;{\mathrm{cm}}^{-1}$). After adding each 0.02-mL increment, the solution was mixed with a glass stirring rod and left to settle for 60 s before the next set of measurements. Each set of TR-NIRS measurements consisted of 100 distributions of time-of-flight (DTOFs) acquired over 30 s.

### 2.3 Animal Model and Experiments

Animal experiments were conducted under an animal care protocol approved by the local ethics committee. All experimental procedures were conducted while the animals were under anesthetic. Anesthesia was induced in an airtight chamber with 5% isoflurane gas and maintained with 2% isoflurane by mask.

**Fig. 1:**
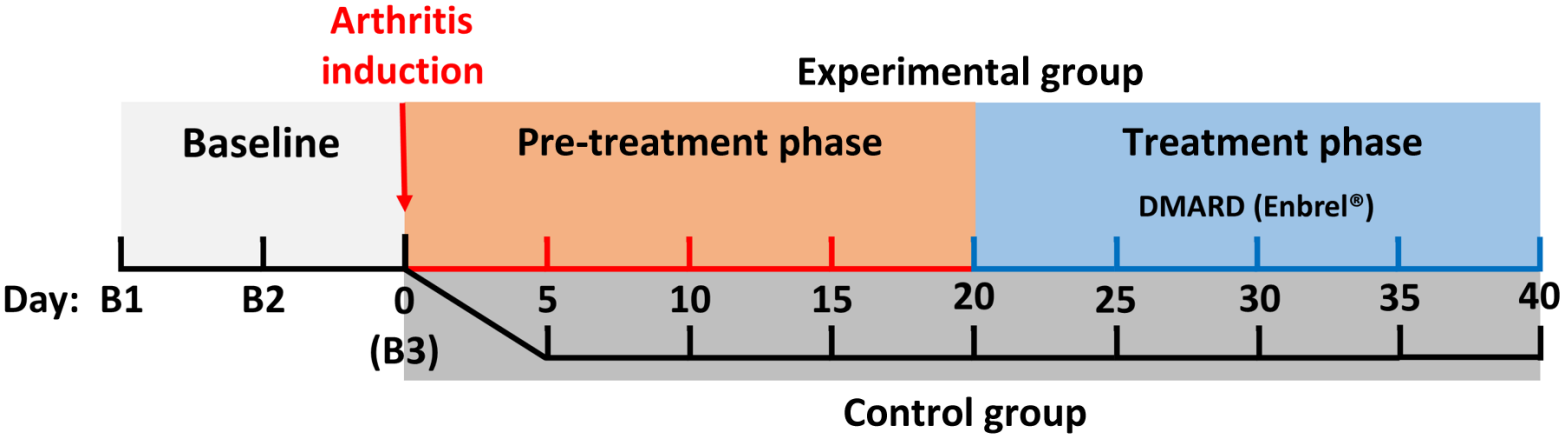
Experimental timeline. Baseline measurements (B1 to B3) were acquired within a 10-day period before and on the day of arthritis induction in the experimental group (day 0). Starting on day 20, animals in the experimental group were treated with the DMARD Enbrel^®^ (etanercept) every 5 days.

The study included 12 adult male Lewis rats: four rats in the control group and eight rats in the experimental group (experimental timeline is shown in Fig. 1). Arthritis was induced using the adjuvant-induced arthritis (AIA) model.^36^ Prior to the start of the study, animals were allowed to acclimate for 5 to 7 days, after which three baseline BF measurements were acquired over a 10-day period (Fig. 1). After the third baseline measurement was acquired on day 0, rats in the experimental group received a subcutaneous injection of a solution prepared using heat-killed lyophilized *Mycobacteria butyricum* suspended in incomplete Freund’s adjuvant to induce polyarticular arthritis;^37^ rats in the control group received saline injections. Thereafter, ankle joint BF was measured every 5 days until the end of the study on day 40 or until rats reached predetermined humane endpoints (HEP) based on pain assessment by a veterinarian. AIA was allowed to progress until day 20 (pretreatment phase). Starting on day 20, the treatment phase of the study began: rats that had not reached HEP were treated with intramuscular injections of the DMARD etanercept (Enbrel^®^: 0.5 mL/kg) every 5 days. Treatments were administered on each measurement day within the treatment phase immediately after BF measurements. Rats in the experimental group were studied in separate cohorts of 2; the entire study protocol (e.g., induction, treatment) was repeated four times with cohorts of 2 rats for a final sample size of 8 in the experimental group. Rats in the control group were studied simultaneously in one cohort of four animals. Weight measurements and qualitative written notes describing animal appearance, behavior, gait, and swelling in the paws or joints were recorded for each rat on and between study days by two certified veterinary technicians (L. Morrison and L. Desjardins). These observations were used in consultation with a veterinarian to determine the appearance of first symptoms of induced inflammation and to assess whether an animal had reached HEP. Evidence of arthritis included visible swelling in paws or joints, weight loss (>5% of initial body weight), reluctance to ambulate, decreased social interaction, and abnormal posture.

**Fig. 2:**
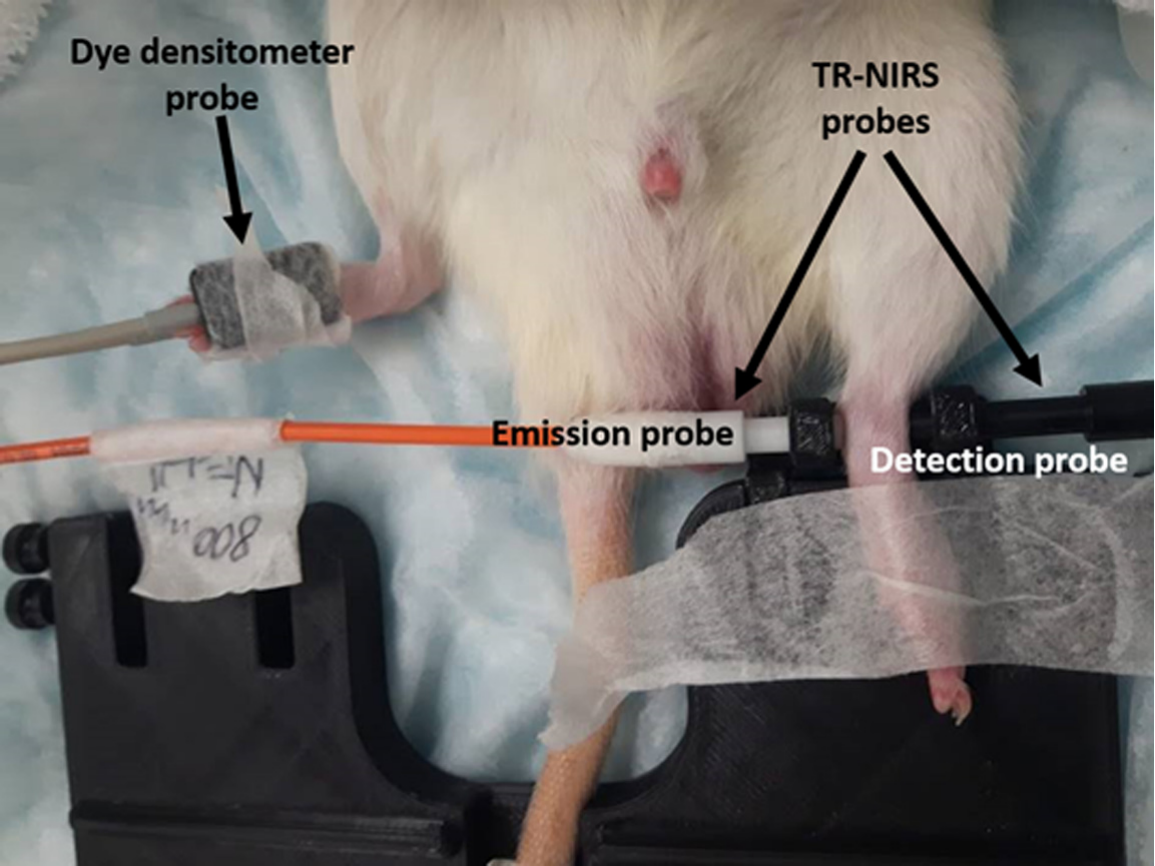
Image of TR-NIRS probe placement on a rat ankle for joint BF measurement. A dye densitometer, placed on the contralateral paw, was used to measure the time-dependent arterial concentration of the BF tracer (ICG) while TR-NIRS probes were used to measure its tissue concentration.

The experimental setup for the BF measurements is shown in Fig. 2. During each measurement, animals received a tail vein bolus injection of the optical contrast agent Indocyanine Green (ICG). A dye densitometer (DDG-2001; Nihon Kohden, Japan) was attached to one paw to measure the arterial concentration of ICG. The dye densitometer also measured the animal’s heart rate (HR) and arterial oxygen saturation, which were recorded and used to assess animal condition throughout the experiment. TR-NIRS measurements were acquired with the emission and detection probes positioned transversely across the rat ankle joint on the contralateral paw (Fig. 2). Each joint BF measurement was started by acquiring 10 s of data before a bolus of ICG solution (0.2 mg/kg) was injected into the rat tail vein. For each BF measurement, we acquired 400 DTOFs over 120 s to obtain the tissue ICG concentration curve. On every measurement day, joint BF was measured on both sides of the animal (i.e., right and left ankles); measurements were repeated twice to mitigate potential unsuccessful measurements. Typical reasons for unsuccessful measurements included data loss due to DDG instrument failure, failed ICG injections due to poor catheter placement, and loss of probe contact (both DDG and TR-NIRS) during acquisition.

### 2.4 Data Analysis

The TR-NIRS measurements were analyzed using an in-house software developed in MATLAB 2017a (The MathWorks Inc., Natick, Massachusetts, 2017). For each measurement, the difference in mean photon time-of-flight (⟨t⟩) between the DTOFs acquired prior to contrast introduction (India Ink for phantom experiments; ICG injection for animal experiments) and the system’s IRF was used to compute the optical pathlength p

$$p=\frac{c}{n}({\left\langle t\right\rangle }_{\mathrm{DTOF}}-{\left\langle t\right\rangle }_{\mathrm{IRF}}).$$ (1)

In Eq. (1), c is the speed of light in vacuum, and n is the refractive index of tissue (n=1.4). The optical pathlength was then used, in combination with the modified Beer–Lambert Law, to determine changes in the absorption coefficient over time [i.e., $\Delta \mu_{a}(t)$]:

$$\Delta \mu_{a}(t)=\ln[\frac{I(t)}{I_{0}}]÷p.$$ (2)

In Eq. (2), I(t) and $I_{0}$ are the detected light intensities after and prior to introduction of the contrast agent, respectively. Note that light intensities were computed as the sum of the total number of photons in each DTOF and that, for animal experiments, changes in pathlength due to ICG injection were deemed negligible as discussed in Sec. 4. For phantom experiments, the measured $\Delta \mu_{a}$ were compared with expected $\Delta \mu_{a}$ for validation. The expected $\Delta \mu_{a}$ were computed using Eq. (3). The molar absorption coefficient of the ink ($\varepsilon_{a,\mathrm{ink}}$) was calculated by determining the molar extinction coefficient $\varepsilon_{e,\mathrm{ink}}$ from a transmission measurement through India ink of known dilution and multiplying it by the ratio $\frac{\varepsilon_{a,\mathrm{ink}}}{\varepsilon_{e,\mathrm{ink}}}=0.885$^38^ as suggested by Spinelli et al:^39^

$$\Delta \mu_{a,\text{expected}}=\ln(10)\varepsilon_{a,\mathrm{ink}}\Delta c,\;\varepsilon_{a,\mathrm{ink}}=0.885\varepsilon_{e,\mathrm{ink}}.$$ (3)

For joint measurements, it was noted that the geometry of the joint, along with its expected structural changes during disease progression, substantially complicates the use of typical analytical solutions of light propagation in tissue and fitting approaches to determine optical properties (see Sec. 4 for detailed discussion). Instead, use of the modified Beer–Lambert Law to compute changes in absorption provides a robust method that is immune to the challenges posed by the joint morphology. Furthermore, the measured $\Delta \mu_{a}(t)$ was converted into an ICG tissue concentration curve using

$$Q(t)=\frac{\Delta \mu_{a}(t)}{\ln(10)\cdot \varepsilon_{\mathrm{ICG}}},$$ (4)

where Q(t) is the time-dependent ICG concentration in the ankle joint, and $\varepsilon_{\mathrm{ICG}}$ is the extinction coefficient of ICG at 805 nm.^40^ The measured tissue and arterial ICG concentration curves were subsequently used to compute joint BF, using a previously reported deconvolution algorithm.^41^ Note that this method of computing BF has been previously tested and validated using phantom and animal experiments.^42^^,^^43^ As mentioned in Sec. 2.3, four perfusion measurements (two per ankle joint) were obtained for each animal in order to account for potentially unsuccessful data collection. If both repeat measurements from one ankle were available, the BF value for that ankle on that day was calculated as the mean of the two measurements. It is noteworthy that in ∼10% of cases, one out of the two repeat measurements was missing and only the nonmissing value was used.

### 2.5 Statistical Analysis

Statistical analysis was conducted using SPSS Statistics 25 (IBM Corp., Armonk, New York, 2017), and power analysis was performed using G*Power software.^44^ For the phantom experiments, agreement between expected and measured changes in $\mu_{a}$ was investigated using linear regression. Regression analysis was performed using a y-intercept fixed at 0 with the assumption that $\Delta \mu_{a,\text{observed}}=\Delta \mu_{a,\text{expected}}=0$ prior to the addition of any India ink. Prior to analysis, regression assumptions of linearity and homoscedasticity were confirmed. For the linearity assumption, a visual inspection of scatter plots of raw observed versus expected values was used to confirm a linear relationship between the two variables. Data homoscedasticity was confirmed by generating scatter plots of residuals versus values predicted by the regression model and visual confirmation of consistent variance of the residuals for all predicted values. Furthermore, assumption of normality in the dataset was confirmed using the Shapiro–Wilk test.

For the *in vivo* experiments, statistical analysis was only conducted for timepoints that contained data from all animals, to account for animals reaching HEP before study completion. Though this approach limited the scope of the statistical analysis, it was necessary in order to avoid any potential survivorship bias within the dataset. A three-way repeated measures analysis of variance (ANOVA) was conducted with time and measurement side (i.e., left or right) as the within-subjects variables, and subject group as the between-subjects variable (i.e., control or experimental group). Prior to analysis, normality of the dependent variable and sphericity were confirmed, using the Shapiro–Wilk test and Mauchly’s test of sphericity, respectively, to ensure no assumptions inherent to ANOVA were violated. In addition, visual inspection of boxplots and histograms of BF data within each group revealed no significant outliers. Upon discovering a significant effect, differences were uncovered using a post-hoc Tukey’s honest significant difference test to account for multiple comparisons. A post-hoc test in G*Power was then used to assess the power of the findings using an effect size equal to the partial $\eta^{2}$ value determined from the ANOVA.

The potentially confounding effects of HR on joint BF were investigated using correlation analysis. Since joint BF could be affected by arthritis induction and DMARD treatment, as well as other temporal factors, the effect of HR on joint BF could only be isolated on a per day basis. Thus, for timepoints with no missing data, each animal’s raw daily BF measurements were correlated with their corresponding HR to generate a correlation coefficient between BF and HR for each day. Since each correlation coefficient was derived using only four comparisons (one for each raw measurement), the resulting coefficients were prone to error and difficult to interpret in isolation; instead, all obtained correlation coefficients were averaged with the assumption that the presence of an underlying trend in the data would skew the average correlation coefficient.

## 3 Results

### 3.1 Phantom Experiments

**Fig. 3:**
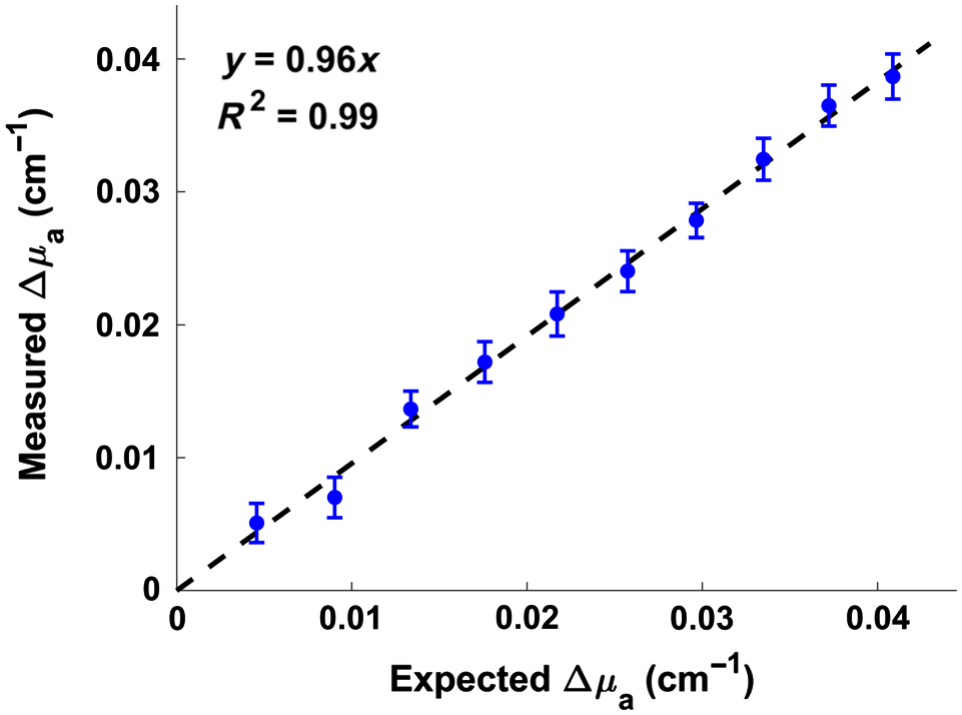
Comparison of expected and measured changes (mean±SD) in the absorption coefficient of a tissue-mimicking solution (0.8% Intralipid) caused by incremental addition of India Ink. One hundred measurements were acquired using the time-resolved NIR spectroscopy system, and absorption coefficients were computed using the modified Beer–Lambert Law. Note that absorption changes measured during subsequent animal experiments were within this range.

A simple linear regression with the y-intercept set to 0 (see Sec. 2.5 for details) was conducted to compare $\Delta \mu_{a}$ measured in tissue-mimicking solution with the expected $\Delta \mu_{a}$ due to India ink addition. There was a significant linear relationship between measured and expected $\Delta \mu_{a}$ [F(1,9)=5773, p<0.05], and a strong relationship between the variables ($R^{2}=0.99$) with a slope of 0.96 within the tested 0.005 to $0.040\;{\mathrm{cm}}^{-1}$ absorption range (Fig. 3).

### 3.2 Animal Experiments

**Fig. 4:**
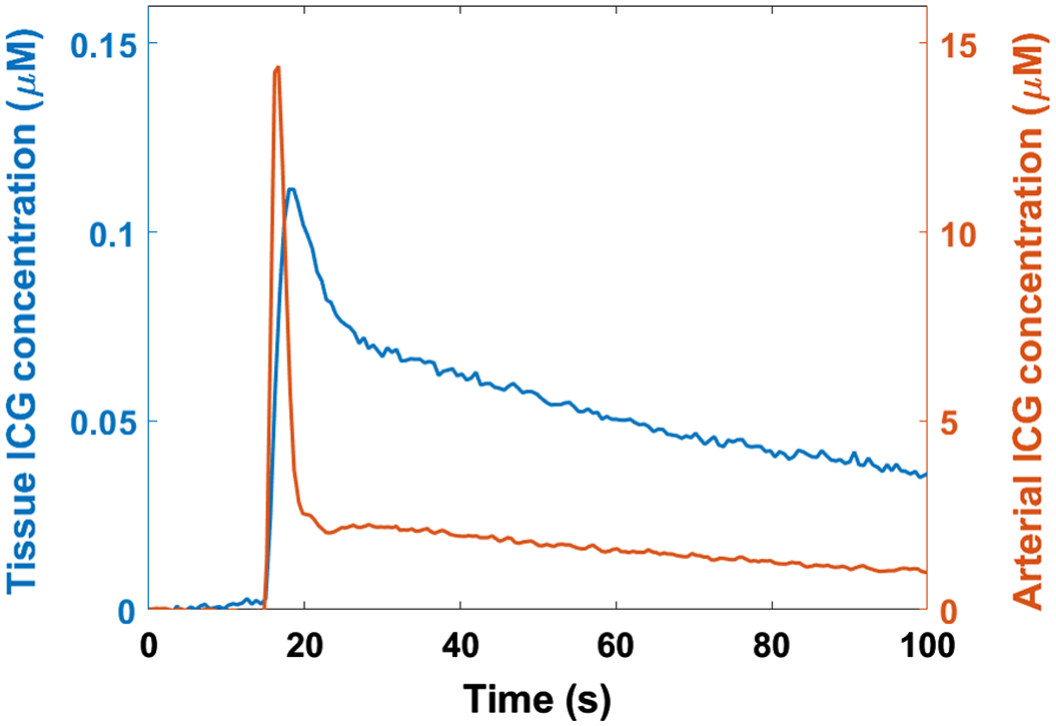
Arterial and tissue ICG concentration curves measured by the dye densitometer and the TR-NIRS system, respectively. ICG was injected into the rat’s tail vein at the 10-s mark.

Figure 4 shows typical arterial and tissue ICG concentration curves measured during an animal experiment. The dynamic inflow and washout of the tracer can be clearly identified on both curves, with the tissue curve showing a slower rise and subsequent clearing of ICG compared with the arterial curve.

**Fig. 5:**
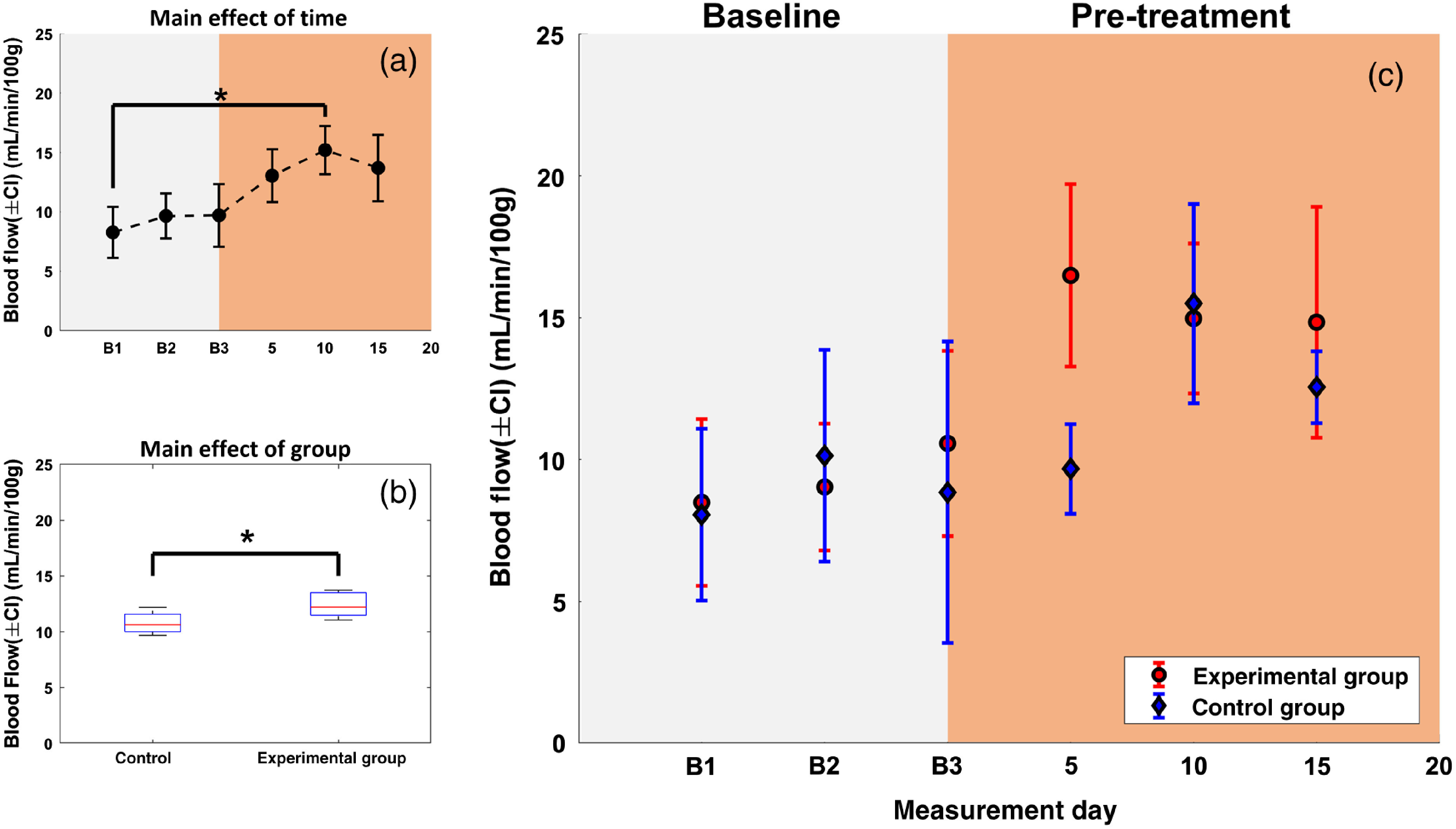
(a) BF (±95% confidence interval) for time courses averaged by group and measurement side, and (b) boxplots of BF for each group averaged over time and measurement side. Asterisks indicate a significant difference at the p<0.05 level between (a) two timepoints or (b) two groups. To facilitate interpretation of main effects, BF (±95% confidence interval) time courses for each group averaged over measurement side are shown in (c). Time courses (a) and (c) show three baseline measurements (B1 to B3; gray), followed by three measurements postarthritis induction in the experimental group (after B3: day 0; orange). Note that, to avoid survivorship bias, statistical analysis was only conducted on timepoints that included all animals in each group; thus, data from the treatment phase are not shown.

The results of the statistical analysis from the first day of the baseline period (B1) to day 15 are summarized in Table 1. In all cases, the BF data were not significantly different from a normal distribution (p=ns; ns, not significant) and did not violate the assumption of sphericity (p=ns). Only time and group were found to have a significant main effect on joint BF (Table 1) with effect sizes of partial $\eta^{2}=.400$ and $\eta^{2}=.365$, respectively. These effects were interpreted as follows: BF increased over time regardless of group assignment, and experimental group BF was higher than control group BF regardless of measurement time. Figures 5(a) and 5(b) show the data relevant to the statistically significant effects denoted in Table 1: a BF time course averaged over measurement side and group, and group BF values averaged over time and measurement side. As seen in Fig. 5(a), a post-hoc Tukey test for the effect of time on ankle joint BF revealed a significant difference between BF on the first day of baseline (B1) and BF on day 10 at the p<0.05 level. In addition, since statistical analysis revealed that measurement side was not implicated in any interaction and did not have a significant main effect on BF (Table 1), time courses for each group averaged over measurement side are presented in Fig. 5(c) to facilitate interpretation of the uncovered main effects. We note that the primary effect of interest in this work was the presence of a two-way interaction between time and group, which would have manifested as a significant difference between the time courses in Fig. 5(c). The presence of this effect would have been interpreted as a difference in joint BF between the experimental and control group over time. To further investigate the lack of this effect (Table 1), post-hoc power analysis was calculated for the two-way interaction between time and group using an effect size of partial $\eta^{2}=0.151$ and yielded a power of 0.21.

**Table 1:** Summary of three-way repeated measures ANOVA with time and measurement side as the within-subject variables, and group as the between-subjects variable. Analysis was conducted on data from the first day of the baseline period (B1) to day 15 postinduction of arthritis.

Variable(s)^a^	F-statistic	p-Value^b^
time^a^group^a^side	F(5,50)=0.149	p=ns
time^a^side	F(5,50)=0.659	p=ns
time^a^group	F(5,50)=1.773	p=ns
group^a^side	F(1,10)=0.135	p=ns
Time	F(5,50)=6.670	p<0.05
Group	F(1,10)=5.736	p<0.05
side	F(1,10)=0.129	p=ns

a Interaction between multiple variables

b ns: not significant

Confounding effects of HR on measured joint BF values were investigated by correlating raw BF measurements with their corresponding HR and averaging across all animals and days as described in Sec. 2.5. This analysis revealed a weak positive correlation (r=0.1370) between HR and BF measurements.

**Fig. 6:**
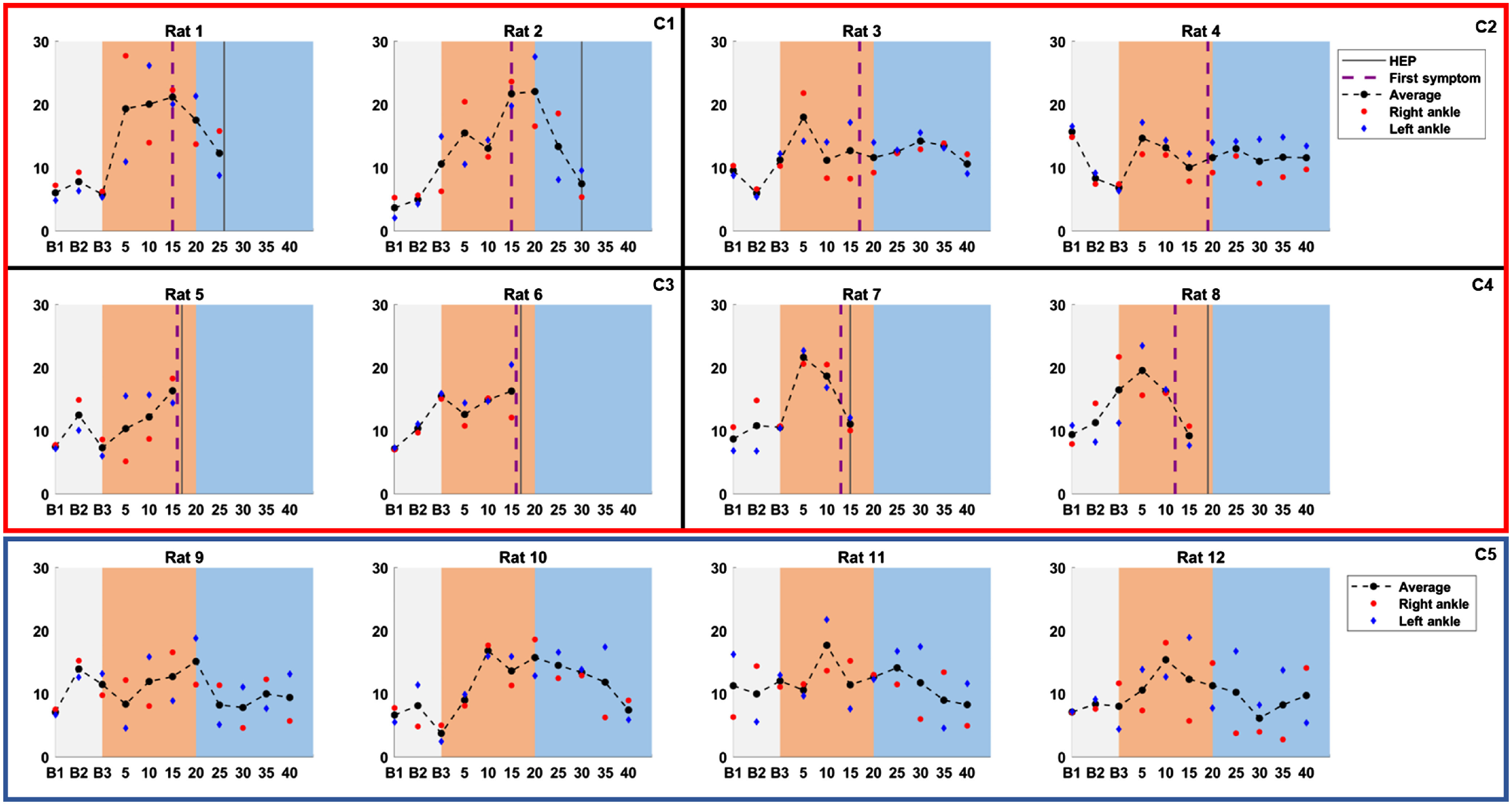
Scatter plots of individual BF values (mL/min/100 g) for each rat; average BF values are shown as black markers, whereas right and left ankle measurements are indicated using red and blue markers, respectively. Time courses show three baseline measurements (B1 to B3; gray), followed by measurements post-arthritis induction in the experimental group (after B3: day 0; orange) and after DMARD treatment was started (days 20 to 40; blue). Experimental and control group data are enclosed by red and blue borders, respectively. For the experimental group, HEP and appearance of first symptoms are indicated by solid and dashed vertical lines. Rats in the experimental group were studied in cohorts of 2 while those in the control group were studied in one cohort of 4; experimental group data are subdivided by black lines to delineate cohorts. Cohort numbers for both groups are indicated at the top right (C1 to C5).

To further investigate the variations in joint BF, longitudinal measurements from each individual rat were examined (Fig. 6); time courses for raw data as well as data averaged across measurement side are presented. Consistently elevated joint BF levels throughout the pretreatment phase were only observed for rats 1 and 2; these animals had the highest incidence of ankle BF above the typical baseline threshold of ∼15  mL/min/100  g. For four out of eight rats in the experimental group, joint BF on day 5 was higher than their typical baseline values. In the control group, joint BF on day 10 for was higher than typical baseline values for three out of four rats. Rats 3 and 4 were the only individuals in the experimental group who completed the entire study period; these rats also showed very minimal variation in their joint BF compared with other rats in their group, and their BF times courses are generally more similar to those of the rats in the control group. In contrast, for rats 1 and 2 joint BF decreased once treatment was initiated; however, only a few treatment timepoints are available since these rats reached HEP quickly. Looking at individual side measurements, there is no clear trend in the inconsistency between measurement sides across animals. For the experimental group, average BF time courses among rats are more similar within each cohort than between cohorts. This similarity is also present in the occurrence of first symptoms and reaching of HEP (Fig. 6).

## 4 Discussion

This work sought to investigate whether joint BF, as measured with DCE TR-NIRS, could track longitudinal changes in joint inflammation during disease induction and DMARD treatment in the AIA rat model of RA. AIA was the first-described animal model of RA and remains widely used for preclinical assessment of RA treatments.^45^ The model is typically characterized by rapid onset (e.g., 10 days postinjection) and progression of polyarticular arthritis; however, the inflammation tends to subside after a month of disease activity. While the AIA rat model does not exhibit the chronic disease progression characteristic of human RA, which takes place over months, it shares many of the relevant biological features, such as swelling, joint destruction, cell infiltration, and T-cell dependence. As well, treatment with etanercept—a common biologic drug that is currently used to treat RA patients—is known to be active in this model and has been associated with slight reductions in arthritic and radiographic scores.^36^^,^^46^ It is important to note that rats tend to develop antibodies against etanercept; thus, when considering the difference in body weight, response to the drug is limited to higher treatment doses (e.g., 4 to 10 mg every 3 to 5 days)^21^^,^^46^ than what is typically used in RA patients (50 mg weekly).

For proper quantification of joint BF, DCE TR-NIRS relies on an accurate measurement of the dynamic $\mu_{a}$ changes in a tissue bed following an ICG bolus injection. These measurements can be used to compute a tissue ICG concentration curve, which can then be combined with an arterial ICG concentration curve to calculate BF. As such, we conducted tissue-mimicking phantom experiments to ensure that our TR-NIRS system could accurately measure changes in $\mu_{a}$. Figure 3 shows a strong linear relationship ($R^{2}=0.99$) with a slope of 0.96 between expected and measured $\mu_{a}$ changes in a tissue-mimicking phantom whose absorption coefficient was modulated using various concentrations of India Ink; these results confirmed our system’s ability to quantify static changes in $\mu_{a}$. Next, the ability of the TR-NIRS system to track dynamic changes in absorption was confirmed through preliminary animal experiments during which representative arterial and tissue ICG concentration curves were obtained. Tissue ICG concentrations were ∼100 times lower than the concentrations of the tracer in arterial blood (Fig. 4), which was similar to what we previously reported for tissue concentration values in the rabbit knee joint.^35^

Following system validation, experiments were conducted in 12 rats: four rats in the study’s control group and eight rats in the AIA experimental group. To account for potential injection effects, rats in the control group received saline injections to match any injections administered to the experimental group. Due to the severity of the AIA model, 50% of rats in the experimental group reached HEP before day 20 postinduction of arthritis. Thus, to avoid survivorship bias, we limited the temporal scope of our subsequent statistical analysis to timepoints that contained data from all animals, i.e., all baseline timepoints and the first three timepoints of the pretreatment phase. The only significant effects found in this study were that both time and group had a significant main effect on joint BF with effect sizes of $\eta^{2}=0.400$ and $\eta^{2}=0.365$, respectively [Figs. 5(a) and 5(b)]. Interestingly, the implication of the main effect denoted in Fig. 5(b) is that BF differed between rats in both groups regardless of when it was measured (i.e., even at baseline). To further investigate this possibility, we compared mean BF time courses of both groups along with their 95% confidence intervals [Fig. 5(c)]; since measurement side did not have a significant effect on BF, we averaged time courses across both ankles to facilitate data interpretation. First, BF appears consistent between both groups and among the baseline timepoints [Fig. 5(c)]; this is an important result as it confirms the ability of our DCE TR-NIRS technique to reliably quantify joint BF. Second, once arthritis is induced (pretreatment), BF in the experimental group is substantially higher than in the control group on day 5 after which BF in the control group rises to match that of the experimental group. These trends in Fig. 5(c) are consistent with the significant effect seen in Fig. 5(a); regardless of AIA, joint BF changes as the study period progresses. Furthermore, Fig. 5(c) suggests that the significant effect described by Fig. 5(b) is largely driven by the discrepancy in BF between both groups on day 5. Nevertheless, this single discrepancy was not substantial enough for the time courses in both groups to be statistically different from one another (i.e., no significant interaction between time and group).

As mentioned in Sec. 1, it is well established that chronic inflammation, which is a key feature of RA, leads to the development of hypoxic regions within the synovium^15^^,^^17^ and that the presence of hypoxia acts as a potent signal for angiogenesis^17^ and increased tissue BF.^19^^,^^20^ Based on this known pathophysiology of inflammatory arthritis and the results from our previous study in a rabbit model of inflammatory arthritis,^35^ we expected to find an interaction between time and group in this study. More specifically, we anticipated an increase in joint BF in the experimental group compared with the control group during the pretreatment phase.

One possible reason that this study did not find a significant difference between control and experimental group time courses was the introduction of measurement error due to the variation in animal HR during different BF measurements. However, only a very weak correlation (r=0.1370) was noted between HR and joint BF, suggesting that the potentially confounding effect of varying HR throughout an experiment had a negligible effect on the BF results. Another reason could be the relatively small sample size. Based on the effect size observed here for the two-way interaction between time and group (partial $\eta^{2}=0.151$), post-hoc power analysis revealed that, in regards to this two-way interaction, our study only had a power of 0.21. To reach a power of 0.8 with the aforementioned effect size, our study would require a sample size of 74; this is a notable difference from the large effect size we previously measured in an inflammatory monoarthritis rabbit model,^35^ where only four subjects were needed to detect a statistically significant difference between control and inflamed joints at the p<0.05 level with a power of 0.8. In particular, this discrepancy between both studies highlights the challenge of modeling human disease with animal models.

Aside from differentiating between groups during the pretreatment phase, this study sought to investigate whether treatment with etanercept affects joint BF in the AIA model postinduction of arthritis. Since etanercept is known to be active in this model of arthritis,^36^^,^^46^ we expected to see decreased joint BF in response to etanercept treatment. However, because no statistical analysis could be performed for timepoints in the treatment phase, we compared treatment response between control and experimental group rats on a case-by-case basis (Fig. 6). Only rats from cohorts 1 and 2 progressed into the treatment phase, and only rats from cohort 1 showed evidence of BF decrease in response to treatment. In particular, their BF dropped from the elevated pretreatment values on days 15 and 20 to values similar to cohort 2 and control rats on days 25 and 30. Rats in cohort 2 did not have elevated pretreatment BF values, and their BF remained relatively constant throughout the study. In fact, aside from a slight elevation in BF in rat 3 on day 5, the time courses of the rats in cohort 2 looked quite similar to those of the control group. Consultation of recorded animal well-being metrics revealed that rats 3 and 4 had the latest appearance of first symptoms (Fig. 6) and, while both rats exhibited some paw swelling, they did not have the gait issues that were typically seen in the other cohorts. Thus, the relatively mild disease induction in cohort 2 rats may have played some role in making it difficult to distinguish between them and control group rats on the basis of joint BF. Importantly, the approach that is typically reported in the literature is to administer treatment on the first day of inflammatory symptoms. In contrast, our study was designed so that treatment was only administered at a set timepoint regardless of first symptom appearance. Since some variability in disease progression is expected and was observed between rats, it is possible that experimental group rats received treatment while in a different state of disease progression, which may have confounded the treatment phase results. Due to these factors as well as the small experimental group sample size available from the treatment phase, the conclusions regarding the relationship between joint BF and DMARD treatment response remain limited.

We also examined the pretreatment phase of Fig. 6 for any consistent differences between the experimental and control groups. Four out of eight rats in the experimental group showed BF increases on day 5, whereas increases from baseline only occurred on day 10 in three out of four rats in the control group; this is consistent with the BF difference on day 5, as shown in Fig. 5(c). A striking observation from Fig. 6 was that, for the entire study period, time courses among rats within the same cohort were more similar than rats from different cohorts. Figure 6 also revealed that rats within the same cohort experienced first symptoms within a day of each other while first symptom occurrence had a standard deviation of 2.20 days when all cohorts were combined. Together, these observations suggest that rats in the same cohort had similar disease progression and that the heterogeneity in the response to arthritis induction between cohorts may have had an important influence on the BF time courses. This observation is further confirmed by the temporal consistency between first symptom appearance and HEP within each cohort. To further investigate this possibility, we used SPSS Statistics 25 to perform a hierarchical cluster analysis on the experimental group’s BF values from the first day of the baseline period (B1) to day 15 postinduction of disease. Since no outliers were present in the dataset, Ward’s method was used as the cluster method and squared Euclidean distance as the similarity metric; the elbow method was subsequently used to identify the optimal number of clusters.^47^ The analysis revealed that four distinct clusters were present in the data: one for each cohort in the experimental group. To confirm the stability of the solution, we randomized the order of the data and reran the analysis three times. In addition, we also performed k-means clustering on the original dataset and assumed that four clusters were present in the data. For all analyses, it was determined that the same four clusters were present in the data, with each cluster corresponding to our original cohort assignments. This provides further evidence that a main source of inconsistency in the study is that animals were divided into small cohorts whose arthritis was induced using four different *Mycobacterium butyricum* solutions. Though every effort was taken to ensure that all solutions were prepared using the same protocol, it is possible that slight variations in injected solution volume or preparation (e.g., mixing of bacteria with Freund’s adjuvant) contributed to varied disease induction among the experimental group.

As shown in this study, temporal variability in intersubject disease and treatment response made it difficult to interpret the link between changes in BF and disease severity. Cases like this benefit particularly from the ability of DCE TR-NIRS to reliably quantify tissue perfusion in absolute units, which makes it possible to compare a single subject’s values over a long range of timepoints to uncover potential physiological relationships. It is important to note that other optical techniques, such as diffuse correlation spectroscopy (DCS), can noninvasively monitor tissue perfusion;^48^^–^^50^ for example, DCS generally provides a BF index whose proportionality to absolute BF is a function of tissue optical properties and tissue geometry. Importantly, RA progression is associated with synovial lining hypertrophy, typically leading to bone resorption, membrane inflammation, edema, and other general morphological changes to the joint tissue, which makes modeling the joint geometry quite challenging.^45^ Considering that these changes vary from subject to subject, accurate quantification of tissue optical properties during disease progression and treatment with DCS could be very difficult. Furthermore, failing to account for these changes would likely confound the relationship between BF indices measured at different timepoints. There are systems that combine DCS with TR-NIRS or frequency-domain NIRS to make it possible to quantify the necessary optical properties for continuous DCS measurement on a day-by-day basis;^48^^,^^51^^,^^52^ however, these approaches still typically rely on the use of analytical solutions to the diffusion equation for simple tissue geometries (e.g., slab geometry). As mentioned in Sec. 2.4, a key advantage of our DCE TR-NIRS technique is that it only requires the ability to quantify changes in absorption to measure joint BF. This circumvents the challenge of needing an analytical solution to the diffusion equation for a joint structure with a complex shape that changes both in time and across subjects and is not well approximated by regular geometrical shapes.

While DCE TR-NIRS has unique advantages that make it suitable for longitudinal joint BF monitoring, a potential source of error while using this technique is the occurrence of dynamic changes in optical pathlength caused by changes in absorption due to the passage of ICG through the joint. To address this, we estimated the change in optical pathlength for five datasets and found that, on average, pathlength varied by ±0.5% over a 120-s measurement period. When these differences were propagated forward in the analysis, they only resulted in a ±1% change in recovered BF values. Since these changes are negligible, for computational efficiency, p was estimated using the mean pathlength measured prior to ICG injection. Another limitation of DCE TR-NIRS is that it requires the use of a contrast agent (e.g., ICG), which may limit clinical translation. However, it has been previously shown that changes in tissue oxy- and deoxyhemoglobin concentrations in muscle following venous occlusion can be used to quantify BF in lieu of a contrast agent.^53^ While the ability of the latter method to quantify joint perfusion has yet to be tested, it offers an interesting alternative that could be readily implemented with TR-NIRS instrumentation and should be an area of future research.

Despite the advantages of the DCE TR-NIRS technique, the small sample size of this exploratory work, issues with animal survivorship, and sources of error linked to disease heterogeneity among the experimental group limit this study’s conclusions regarding the link between BF, arthritis induction, and DMARD treatment response in the AIA model. Overall, we found the use of the AIA model in a continuous, longitudinal study design challenging due to the severe and rapid onset of the disease, which often occurred in the 5-day periods between subsequent BF measurements. We also conducted a subset of experiments to investigate mycobacterium dose and disease severity but could not determine a reliable dose for inducing slower and milder arthritic progression. Considering the mismatch between this relatively rapid disease progression and the relatively low overarching temporal resolution of our data (i.e., one measurement every 5 days), our time courses may have missed otherwise important variations in BF and disease activity. While the temporal frequency of our BF measurements was part of the original study design, which was formulated based on results from our previous study,^35^ it was ultimately a limitation of the study presented here and should be amended in future work. The authors also acknowledge the potentially confounding effect of anesthesia induced by isoflurane on BF in rodents.^54^^,^^55^ Though anesthetic concentrations were tightly managed to consistent levels during experiments, future work may benefit from investigating whether varying isoflurane concentrations or using other anesthetics such as ketamine has an effect on joint perfusion. Future work would also benefit from exploring longitudinal BF changes in milder models of RA with slower symptom onset, such as pristane-induced arthritis,^56^ to allow a more robust investigation of the relationship between DMARD treatment and joint BF.

## 5 Conclusion

In this work, we used our quantitative joint perfusion technique (DCE TR-NIRS) to monitor joint BF in a longitudinal rat model of RA. Joint BF was measured in each animal’s left and right ankles before treatment (pretreatment phase) and after treatment with the DMARD etanercept (treatment phase) in an experimental group of eight rats with AIA. Four additional rats served as controls throughout the study. Time and group had separate significant effects on joint BF; however, there was no significant interaction between time and group despite a large difference in average BF values on day 5. Measurement side did not have a significant effect on joint BF. Statistical analysis of the treatment phase was limited since 50% of the animals reached HEPs before starting treatment. Comparison of individual animal time courses between the experimental and control group revealed no consistent trend in treatment response; effects may have been masked by heterogeneous disease response to AIA in the experimental group. Future work will focus on exploring longitudinal BF changes in milder models of RA with slower symptom onset to allow a more robust investigation of the relationship between DMARD treatment and joint BF.
